# Case of Malignant Tumor Cured by Dietetic Means

**Published:** 1850-02

**Authors:** 


*Case of Malignant Tumour cured by dietetic means.*—"A man about 40 years of age consulted Dr. Twitchell, of Keene, N. H., in relation to a tumour on his scapula, as large as a pint bowl. It was evidently osteo sarcoma, had its usual crackling feel, and resembled very closely one in the same position which Dr. Twitchell had seen a short time previously, and for which he had removed the whole upper extremity, even scapula and clavicle. In that case, the wound healed, but the man died a year or two afterwards, with carcinoma of some internal organ. When the second case applied for advice, Dr. T. declined an operation, and the man returned home to Vermont. Soon afterwards he heard of somebody in New York who could cure him, and applying to this person for advice, received the following:

"He was to take from the brook which ran through his native farm a plant which grew there (the adviser did not know what it would be), and use a weak infusion of it for his only drink every day until the tumour had disappeared. *His diet, besides this, was to consist of bread alone.* This advice was strictly followed—the plant he used was 'water dock.' Dr. Twitchell happened to see the man two years afterwards, when he was still following this course. He found the tumour had nearly disappeared, there being apparently only a trifling thickening of the skin."—*Boston Med. and Surg. Journal, communicated by H. I. Bowditch, M. D., of Boston, Mass.*

